# Strain-controlled power devices as inspired by human reflex

**DOI:** 10.1038/s41467-019-14234-7

**Published:** 2020-01-16

**Authors:** Shuo Zhang, Bei Ma, Xingyu Zhou, Qilin Hua, Jian Gong, Ting Liu, Xiao Cui, Jiyuan Zhu, Wenbin Guo, Liang Jing, Weiguo Hu, Zhong Lin Wang

**Affiliations:** 1 CAS Center for Excellence in Nanoscience, Beijing Key Laboratory of Micro-nano Energy and Sensor, Beijing Institute of Nanoenergy and Nanosystems, Chinese Academy of Sciences, Beijing, 100083 China; 2 School of Nanoscience and Technology, University of Chinese Academy of Sciences, Beijing, 100049 P. R. China; 3 Graduate School of Electrical and Electronic Engineering, Chiba University, Chiba, 263-8522 Japan; 4 Estuarine and Coastal Environment Research Center, Chinese Research Academy of Environmental Sciences, Beijing, 100012 P. R. China; 5 Center on Nanoenergy Research, School of Physical Science and Technology, Guangxi University, Nanning, 530004 China; 6 School of Materials Science and Engineering, Georgia Institute of Technology, Atlanta, GA 30332-0245 USA

**Keywords:** Electrical and electronic engineering, Electronic devices, Electronic and spintronic devices

## Abstract

Bioinspired electronics are rapidly promoting advances in artificial intelligence. Emerging AI applications, e.g., autopilot and robotics, increasingly spur the development of power devices with new forms. Here, we present a strain-controlled power device that can directly modulate the output power responses to external strain at a rapid speed, as inspired by human reflex. By using the cantilever-structured AlGaN/AlN/GaN-based high electron mobility transistor, the device can control significant output power modulation (2.30–2.72 × 10^3^ W cm^−2^) with weak mechanical stimuli (0–16 mN) at a gate bias of 1 V. We further demonstrate the acceleration-feedback-controlled power application, and prove that the output power can be effectively adjusted at real-time in response to acceleration changes, i.e., ▵P of 72.78–132.89 W cm^−2^ at an acceleration of 1–5 G at a supply voltage of 15 V. Looking forward, the device will have great significance in a wide range of AI applications, including autopilot, robotics, and human-machine interfaces.

## Abstract (web summary)

Designing intelligent power devices that can directly control the output power modulation responses to external stimuli at a rapid speed remains a challenge. Here, the authors report a strain-controlled power device by using the cantilever-structured AlGaN/AlN/GaN HEMT to emulate human reflex process.

## Introduction

The tremendous developments of artificial intelligence (AI) have occurred and dramatically changed our life, such as intelligent robots and autopilot technologies^1,2^. Nature has provided us with large numbers of inspiring examples to inspire new devices for emerging AI applications^3,4^. Recently, significant efforts have been made in developing bioinspired functionalities to devise intelligence devices or systems^1,5^, e.g., e-skin^6^, e-nose^7^, cochlear^8,9^, prosthesis^10^, and larynx^11^.

In practical applications, conventional sensor-actuator systems (e.g., pressure sensors) typically employ sensitive components and varistors for transducing mechanical signals (e.g., displacement, velocity, and acceleration) into electrical signals (e.g., voltages, and currents). However, the transduction process inevitably requires complex circuits modules, including analog-to-digital (A/D) or digital-to-analog (D/A) converter, strong/weak electricity isolation, and CPU intervention. To date, AI is primarily based on programming and relies on computer-controlled electronic devices^12,13^, which is also called unsupervised condition. Such AI applications in a self-driving car and robotic posture balance need to be able to perform real-time control of output power at a rapid speed, according to changes in external conditions. Besides, it is also essential for people to ultimately perform terminal control, which is called supervised condition. Remarkably, power devices that can directly realize the output power modulation responses to external stimuli at unsupervised/supervised conditions are very highly desirable for practical use of future AI technology. However, power devices used in AI systems, achieving automate actions and manual manipulate equipment in unexpected environmental changes, is a long-term challenge^14–16^. Driven by this challenge, people have devoted many efforts to developing new intelligent power devices from bioinspired forms^2,17^.

III-V materials alloys with semiconductor and piezoelectric properties are extremely promising for power electronics applications^18,19^. Among various materials or devices, the AlGaN/AlN/GaN high electron mobility transistor (HEMT) shows the great potential in power electronics, due to its high carriers density, high mobility, and large breakdown feild^20,21^. Based on the piezoelectric characteristics of the AlGaN/AlN/GaN heterojunction, the static piezoelectric charge generated at the interface under strain can be converted into a corresponding change in the two-dimensional electron (2DEG) density formed at the interface^22–24^. In recent years, some groups^18,25–27^ reported interesting results that strain-induced piezoelectric polarization charges presented at the local interface can be used to modify the 2DEG concentration, as a result of the piezotronic effect. And the piezotronic effect, coupling the semiconductor and piezoelectric properties, is typically applied to modulate the electric transport properties of nanowires^18^, sensors^28^, and HEMTs^21^. Moreover, the AlGaN/AlN/GaN-based piezotransistive cantilever, which makes HEMT in the cantilever architecture, is an attractive way to dramatically improve its output power density by driving strain at the free-end, as a result of manipulating the piezoelectric polarization in the heterojunctions. That is, the conductivity of the piezotransistive cantilever is directly changed by external strain^29^. Thus, it is a building block for establishing a seamless, sensitive, and real-time interaction between the output power and external stimuli.

Here we present a strain-controlled power device (SPD) that is capable of using external strain modulating the output power by emulating the human reflex process. The output power characteristics of the SPD under strain are systematically investigated. The SPD exhibits an ultra-high-output power with a sensitive piezotronic modulation. The external strain can modulate the output power density, while the gate voltage can take the ultimate control of the output power, which means that the strain-modulated-power is programmable. Additionally, the flexible and miniaturized device also provides additional freedoms for complex motion and adaptation to environmental constraints. More impressively, we also demonstrate the potential application of the SPD in acceleration-feedback-control. Moreover, this work provides not only insights into interactions of mechanical stimuli and power control, but may also promote the development of intelligent power devices similar to human reflex. The SPD will have excellent application prospects to domains ranging from automotive to biomedical, such as automatic driving, robotic control system, and human-machine interfaces.

## Results

### Human reflex inspired SPD

**Fig. 1:**
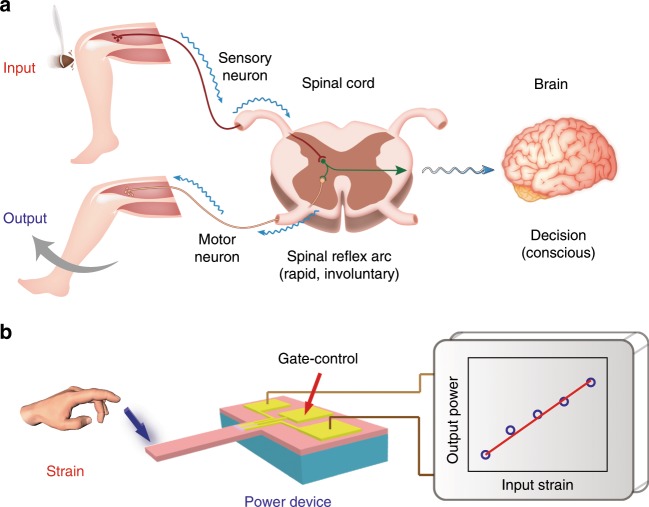
Concept of strain-controlled power device (SPD) as inspired by human reflex. **a** Schematic illustration of human reflex. When a stimulus is received from a thigh muscle at the knee, the action potential in the sensory neuron is sent to the gray matter of the spinal cord. The sensory neuron in the spinal cord directly establishes a synaptic connection with the motor neuron. If the signal is strong enough, it can trigger action potentials in motor neuron, causing knee reflex. Moreover, knee reflex is a spinal reflex whose reflected nerve center is in the spinal cord, but is still modulated by the high central nervous system (brain). **b** Schematic illustration of the SPD. The external strain applied to the cantilever-based power device can emulate the logic process of human reflex to control the output power with the input mechanical stimulus.

The knee-jerk reaction of human reflex has fascinated scientists due to its unique characteristics, such as the ability to slam signals to output bouncing signals through the gray matter of the spinal cord and ability of the brain to control knee reflexes during a crisis. Figure 1 illustrates the biological model for human reflex, and the bioinspired electronic counterpart, i.e., the SPD. The reflex action is a rapid and involuntary movement in response to a stimulus, which does not receive or need conscious thought. As schematically illustrated in Fig. 1a, when a stimulus is received from a thigh muscle at the knee, a neural impulse (action potential) in the sensory neuron is sent to a synapse in the spinal cord but not directly into the brain. The sensory neuron directly establishes the synaptic connection in the spinal cord with the motor neuron. If the signal is strong enough, it can trigger action potential in the motor neuron, causing knee reflex. The reflex signals occur very quickly via the synapse in the spinal cord without the routing delay through the brain, but it is can be intervened by the brain in some cases. In short, the knee reflex is a spinal reflex arc that reflects an automatic response to a stimulus directly through the low central nervous system—spinal cord in a rapid and involuntary manner (i.e., unsupervised condition). Meanwhile, it can be still modulated by the high central nervous system - brain (i.e., supervised condition).

In the conventional sensor-actuator system, the power devices are typically operated through a complex series of processes, including A/D or D/A converter, strong and weak electrical isolation, and CPU control (Supplementary Fig. 1). The chain sensor-actuator system is complex, lengthy, and unstable. As inspired by the biological reflex and its working mechanism, the SPD is represented to directly modulate the output power by applying external strain through the simple cantilever-structure design, which greatly simplifies the system complexity. An external strain is loaded on the SPD which emulates the reflex process of mechanical stimuli. As shown in Fig. 1b, the strain induces a deflection of the SPD which leads to a change of 2DEG concentration at the interface of the AlGaN/AlN/GaN heterojunctions, and then directly responses to the output power density without any delay induced by the conventional sensor-actuator system (i.e., unsupervised condition). More importantly, the gate bias (*V*_gs_) can be used to take the ultimate control of modulating output power level, which is analog to the intervention of the brain (i.e., supervised condition). Different from the conventional sensor-actuator system, the SPD has high-power output and is capable of exhibiting the output modulation at weak mechanical stimuli with real-time, compact, and stable responses.

### SPD design

**Fig. 2:**
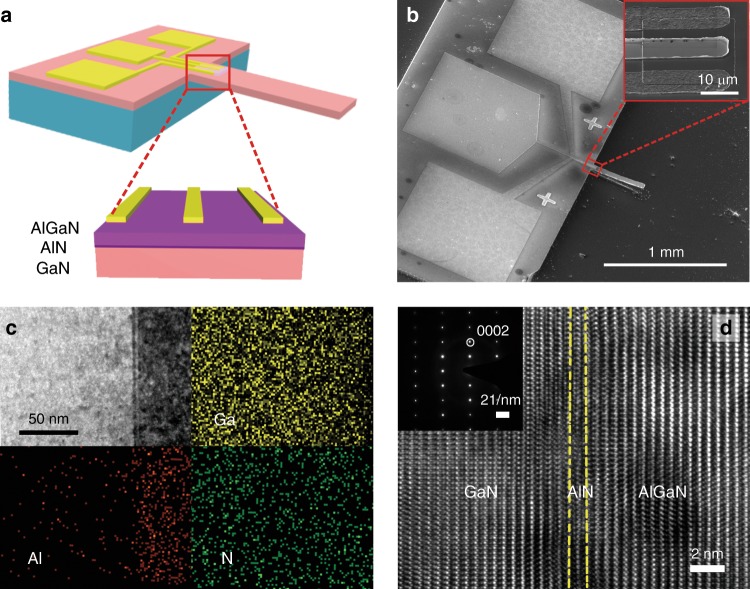
Characterizations of the SPD. **a** Schematic illustration of the SPD with the AlGaN/AlN/GaN heterostructures. **b** Scanning electron microscopy (SEM) image of the SPD. The inset shows a magnified section containing the AlGaN/AlN/GaN-based HEMT. **c** Cross-sectional high-angle annular dark-field scanning transmission electron microscope (HAADF-STEM) image of the AlGaN/AlN/GaN hetero-stacks in the SPD, and the corresponding energy-dispersive X-ray spectroscopy (EDS) element mapping including Ga, Al, and N. **d** High-resolution TEM image acquired from the AlGaN/AlN/GaN hetero-stacks. The inset is the corresponding selected area electron diffraction (SAED) of GaN.

The SPD is designed by using the AlGaN/AlN/GaN heterojunction in a cantilever architecture, as schematically shown in Fig. 2a. The enlarged part is the schematic cross-section of the AlGaN/AlN/GaN heterostructure. The thicknesses of AlGaN, AlN, and GaN layers are 30 nm, 1 nm, and 4.3 μm, respectively. The detailed fabrication process is described in the Method and Supplementary Fig. 2. The cantilever-based structure of the as-fabricated SPD is clearly shown in the scanning electron microscopy (SEM) images of Fig. 2b. And the inset illustrates the geometry of gate and source-drain contacts. We develop a fully dry etching process by using the inductively coupled plasma etching (ICP) to fabricate the GaN-based cantilever, and the details of the ICP-based dry etching steps are schematically shown in Supplementary Fig. 3. The two key procedures are illustrated as follows: (i) trenches are fabricated by anisotropic etching of GaN/Si (Supplementary Fig. 3b); (ii) the cantilever structure is laterally released by isotropic etching of Si (Supplementary Fig. 3c). The anisotropic/isotropic etching steps can be easily controlled by simply adjusting the etching recipe (e.g., gas mixture, power, and time). The presented fully dry etching process has many unique advantages including precise control, well-aligned shape, less-contaminated, and easily-integrated into Si-based circuits.

The high-angle annular dark-field scanning transmission electron microscopy (HAADF-STEM) images (Fig. 2c) exhibit the AlGaN/AlN/GaN heterostructure. The energy-dispersive X-ray spectroscopy (EDX) elemental mapping (Fig. 2c) further confirms the existence and corresponding distributions of the elements of Ga, Al, and N. And the detailed structural information of the AlGaN/AlN/GaN heterostructure is investigated by high-resolution transmission electron microscopy (HRTEM) and selected area electron diffraction (SEAD) (inset of Fig. 2d). The interfaces atoms of AlGaN/AlN and AlN/GaN are uniform and sharp without apparent boundary defects or dislocations. The layers of GaN, AlN, and AlGaN can be easily identified, corresponding to the (0002) plane. Additionally, the corresponding line profile extracted from EDX mapping is presented in Supplementary Fig. 4, confirming the variation of chemical compositions.

### Performance of SPD

**Fig. 3:**
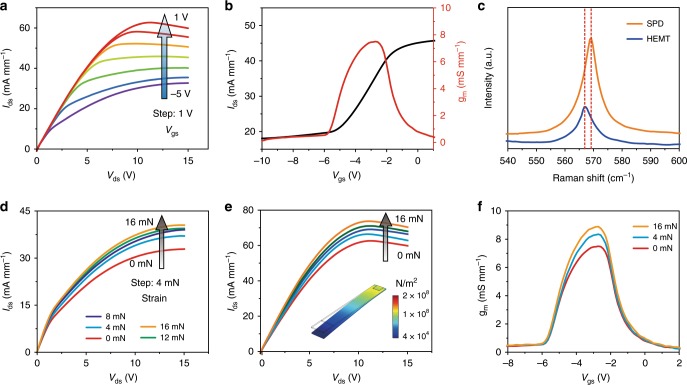
Electrical performance of the SPD. **a** Output (*I*_ds_–*V*_ds_) characteristics of the SPD as the *V*_gs_ ranging from −5 V to 1 V at a step of 1 V. **b** Transfer (*I*_ds_–*V*_gs_) characteristics and the transconductance of the SPD measured at *V*_ds_ = 6 V. **c** Spatial Raman spectra of the AlGaN/AlN/GaN heterostructures before (HEMT) and after (SPD) dry-etching for the cantilever structure. **d**, **e** Output characteristics of the SPD under external strain from 0–16 mN, with the gate voltage *V*_gs_ of (**d**) −5 V and (**e**) 1 V, respectively. The inset of (**e**) illustrates the strain distribution of the SPD under an external strain of 16 mN, which is simulated by COMSOL Multiphysics. **f** The transconductance characteristics of the SPD under various external strain (0 mN, 4 mN, and 16 mN).

The measured I-V characteristics of the source-drain and gate contacts of the SPD and the HEMT are shown in Supplementary Fig. 5a, b and Supplementary Fig. 6a, b, respectively. Both the SPD and the HEMT exhibit typical Ohmic and Schottky contacts. Based on the suitable contacts, the output characteristics (*I*_ds_–*V*_ds_) of the SPD and the HEMT show good capability of gate-control, as respectively shown in Fig. 3a and Supplementary Fig. 6c. The output characteristics show a distinct linear region at a low source-drain bias (*V*_ds_), and then the drain current approaches saturation with further increasing of the drain bias. Large output currents are achieved in both SPD and HEMT and can be effectively controlled at various *V*_gs_ of −5 V to 1 V. When compared to the HEMT, the SPD shows a reduced current of 60 mA mm^−1^ at 10 V because of its suffering from strain partial release and more dry etching process.

Furthermore, the transfer (*I*_ds_–*V*_gs_) characteristics of the SPD and the HEMT at V_ds_ = 6 V are shown in Fig. 3b and Supplementary Fig. 6d, respectively. The maximum transconductance (g_m,max_) of 7.5 mS mm^−1^ is measured in the SPD (Fig. 3b), while the value for the HEMT can reach 52 mS mm^−1^ (Supplementary Fig. 6d). Additionally, Fig. 3c shows the Raman spectra of the SPD and the HEMT obtained at room temperature. Compared with the HEMT, the SPD exhibits that the E_2h_ mode of GaN shows a blue shift from 566.8 to 569.2 cm^−1^. It reveals that the etching process induces a tensile strain relaxation of the GaN layer^30^. The lattice mismatch between III-nitrides and silicon substrate can lead to a high tensile strain. Moreover, the lifting-off process removes the silicon substrate to partially release the tensile strain, which weakens the piezoelectric polarization to decrease the concentration of 2DEG at the AlGaN/AlN/GaN interfaces. In addition, defects will be introduced during the complex etching process of the SPD, which also degrades the electrical performance to some extent.

### Piezotronic effect of SPD

Based on the piezotronic effect, the output power of the SPD can be effectively modulated by external mechanical stimuli in real time. The external strain is loaded on the free-end of the cantilever by using a probe needle loaded along the c-axis direction. An increase in in-plane tensile strain occurs in the AlGaN/AlN/GaN heterostructure. The deflection depth of the cantilever can be tuned by the probe needle in the controllable/reproducible manners. And the loaded strain can be calculated with equations described in Supplementary Note 1. And thus, the normal force increases from 0 to 16 mN as the deflection depth of the cantilever increases from 0 to 20 μm.

In order to further understand the strain effect on the output characteristics of the SPD, different external strains are loaded on the free-end of the cantilever. It can be clearly observed that the *I*_ds_–*V*_ds_ curves show up-shifts to some degrees according to the increasing of the strain, which are both shown in Fig. 3d, e. It means a direct and effective output power modulation by a weak mechanical stimulus. As applying an external strain along *c*-axis, a piezo-potential is generated, resulting in the change of 2DEG and thus modulating electron transport. It should be noted that the SPD is a high-power device capable of directly controlling electricity rather than a strain sensor.

In addition, the output current response to the external strain is also effectively modulated by the *V*_gs_. The controllable capabilities of various *V*_gs_ (−5 V ~ 1 V) under strains of 0 ~ 16 mN are presented in Fig. 3d, e and Supplementary Fig. 7a–e, respectively. It can be clearly seen that a progressively higher modulation of current density is directly controlled at a *V*_gs_ of 1 V with the same strain. When the cantilever is subjected to an external strain, the output current density of the cantilever increases, both in the linear region or the saturation region. The saturated current at *V*_ds_ = 15 V reaches 40.43 mA mm^−1^ under the maximum applied pressure (or strain of 16 mN) compared to the SPD without strain (32.83 mA mm^−1^) at *V*_gs_ = −5 V. In contrast, the output current can be reached 70.36 mA mm^−1^ compared to the SPD without strain (59.87 mA mm^−1^) at *V*_gs_ = 1 V. It indicates that the SPD sensitivity is programmable. The inset of Fig. 3e illustrates the strain distribution of the SPD under an external strain of 16 mN, which is simulated by COMSOL Multiphysics. Besides, the transconductance of the SPD at different applied external strain is displayed in Fig. 3f. The transconductance shows an increase with the increasing of applied external strain, indicating that the gate has an increasingly stronger capability to control the channel current. Therefore, it proves that the piezotronic effect, i.e., strain-controlled behavior, can effectively modulate the output characteristics of the SPD.

### Strain-controlled output power

**Fig. 4:**
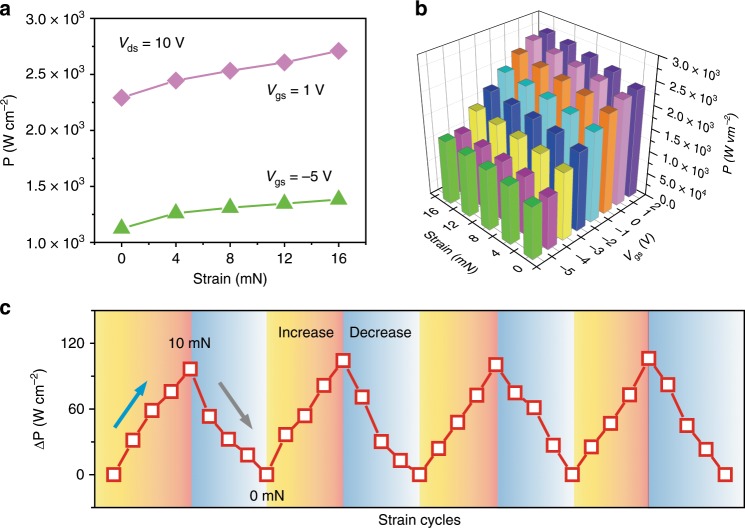
Strain-controlled output power. **a** Output power density plots of under external strain from 0 to 16 mN at the gate voltage of −5 V and 1 V. **b** 3D plots illustrating the relationship between the output power density and the input strain or gate voltage. **c** Reproducible procedures of loading or unloading strain (0–10 mN) in response to the relative output power density in cyclic tests.

The output power modulation of the SPD is further discussed. Figure 4a shows the relationship between output power density and different strain. The output power density dependence on the applied strain can be obtained by employing strain-controlled output characteristics. More specifically, the output power density shows an increase with the applied strain (0–16 mN), as a result of the increasing of the additional electrons and concentrations of 2DEG suffering from in-plane tensile strain. The maximum output power density of the SPD can reach 1.39 × 10^3^ W cm^−2^ and 2.72 × 10^3^ W cm^−2^, respectively, in response to the *V*_gs_ of −5 V and 1 V under the strain of 16 mN. Furthermore, the output power level can also be tuned at the different *V*_gs_, as shown in Fig. 4a, b, and Supplementary Fig. 7f–j. Upon the strain of 16 mN, the relative output power density increases up to 1.51, 1.74, 2.02, 2.29, and 2.54 × 10^3^ W cm^−2^, respectively, at different *V*_gs_ (Supplementary Fig. 7f–j). To check the sensitivity of the output power under various *V*_gs_, the *V*_gs_ sweeps are systemically conducted. The relationships of the output power characteristics of the SPD on the external strain and the *V*_gs_ are all shown in Fig. 4b, illustrating that the output power intensity rapidly increases as the external compressive strain increases. It is due to the increase in 2DEG density with external strain, leading to a significant piezoelectric effect. The results also show that the output power variations are sensitive to the continuous increase of gate voltage in the SPD (Fig. 4b). It means that the *V*_gs_ can significantly change the sensitivity of the output power to external strain. Similar to human reflexes, external stimuli (e.g., strain) can induce knee reflex (analogy to output power changes). Moreover, the brain (gate) reserves the ultimate control over knee reflex. Besides, the reproducible procedures of loading or unloading multiple strains in response to the relative output power density of the SPD are shown in Fig. 4c in cyclic tests, showing very good stable and repeatable performance for the strain-controlled devices.

### Reproducible strain-dependent analysis

**Fig. 5:**
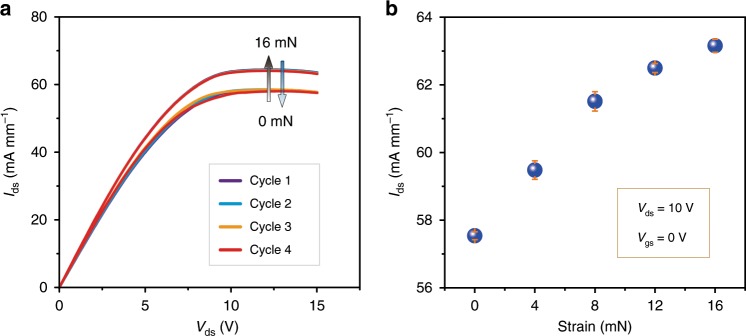
Reproducible strain-dependent analysis of the SPD. **a** Output (*I*_ds_–*V*_ds_) characteristics of the SPD in four consecutive cycles as the load and release of strain (0/16 mN). **b** Statistical variation of the SPD. The data are measured at *V*_ds = _10 V, *V*_gs_ = 0 V under external strain of 0–16 mN. Error bars indicate standard deviations for four sets of data points.

To further investigate the reproducibility of the SPD under external strain, the output currents can be obtained with the same batch sample after repeated loading/releasing strains (0, 4, 8, 12, and 16 mN) at a *V*_gs_ of 0 V. The strain-dependent output current curves and the statistical variation are shown in Fig. 5a, b, respectively. Highly stability and repeatability of the SPD are clearly observed under each external strain (Supplementary Fig. 8). In addition, there is no obvious hysteresis in the strain response procedures, as shown in Fig. 5a. With the accretion of strain (0–16 mN), the output current of the SPD shows a positive correlation (Fig. 5b). And the standard deviations under each strain (0, 4, 8, 12, and 16 mN) are calculated as 0.11%, 0.27%, 0.29%, 0.13%, 0.19%, respectively. The SPD exhibits good reproducibility under the external strain stimuli, which is very suitable for strain-controlled power electronics.

### Strain-responding mechanism

To rationalize the experimental results and further explore the physical mechanism of the SPD, a self-consistent calculation model based on the Schrödinger, Poisson and piezoelectric constitutive equations has been fully developed to simulate the change of 2DEG concentration under external strains. The calculated conduction band (*E*_c_) of the AlGaN/AlN/GaN heterostructure is shown in Supplementary Fig. 9a. And the enlarged *E*_c_ of the AlGaN/AlN and AlN/GaN heterojunctions are shown in Supplementary Fig. 9b, c, respectively. It is obvious that, as the increase of compressive strain on the cantilever, the E_c_ of AlGaN is lifted up while the E_c_ of GaN is lowered down, which will deepen the potential well of the AlN/GaN heterojunction. Owing to the reformation of *E*_c_, the distribution of carrier concentration is calculated and, as a result, varies with external strains (Supplementary Fig. 9d). It can be found that the peak value of carrier concentration increases with strain, indicating that more electrons are confined in the AlGaN/AlN/GaN potential well. By virtue of the semiconductor physics theory, the 2DEG sheet carrier concentration under various strains is obtained by integrating the carrier concentration distribution along the *c*-axis. Supplementary Fig. 9e shows that the 2DEG sheet carrier concentration has an increase with the loading strains ranging from 0 to 16 mN, which contributes to the strain-responding output characteristics and power densities consequently. The calculated 2DEG sheet carrier concentrations qualitatively match well with the experimental results (Fig. 3d), where the *I*_ds_ is proportional to the 2DEG concentration.

### Acceleration-feedback-control

**Fig. 6:**
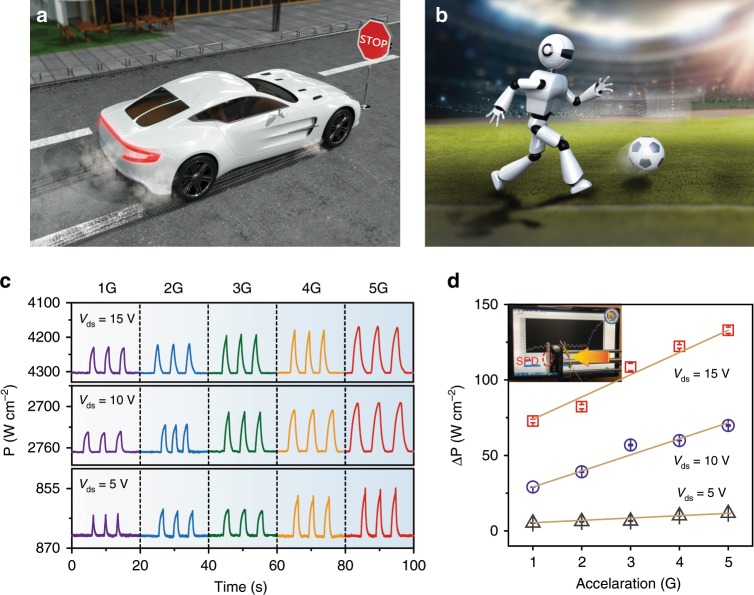
Acceleration-feedback-control in the SPD. **a** Schematic illustration for the self-adjusted, unsupervised output power of emergency braking in a self-driving car. **b** Schematic illustration for the self-regulation of posture balance in robot motions, e.g., soccer robot. **c** Self-regulation of the output power density at real-time in response to the acceleration of 1, 2, 3, 4, and 5 G. **d** The changes in output power density with various acceleration. Error bars indicate standard deviations for three sets of data points. The inset shows the setup for the acceleration-feedback-control.

One of the critical parts of the field of AI is to realize the accelerate-feedback-control. Emerging AI applications, such as autopilot or robot walking, require effectively control output power according to the changes of environment, e.g., acceleration^31–35^. In a self-driving car, the output power of engine needs to be realized at the self-adjusted and unsupervised conditions in rapid response to emergency braking or other accidents, as schematically shown in Fig. 6a. In an advanced robot, the output power should be self-regulated to drive its posture balance at more intelligent manners (Fig. 6b). Acceleration is a typical key parameter for self-driving car or robotic motion, and the accelerate-feedback-control is needed to be further investigated. A new design concept based on SPD is proposed, which uses acceleration to directly control the output power.

The acceleration-feedback-control measurement is performed by using a combined system of semiconductor characterization tester and linear motor. The source-drain voltage (*V*_ds_) varies from 5 to 15 V while the *V*_gs_ is 0 V for the operation. The SPD has a fast response and recovery switching. Figure 6c shows the power density as a function of the different acceleration. It shows an excellent sensitivity on the acceleration of reciprocating motion from 1 to 5 G. A significant decrease in output power density is clearly observed as the acceleration increases, and the achievable power densities under different *V*_ds_ (5, 10, and 15 V) at an acceleration of 5 G are 866.8, 2765.9, and 4304.2 W cm^−2^, respectively. The excellent power response performance is attributed to the high-quality AlGaN/AlN/GaN heterojunction in an advanced SPD structure. We also demonstrate the self-regulation of the output power density of the SPD at real-time in response to the acceleration of 1, 2, 3, 4, and 5 G at a *V*_ds_ of 1 V, and the detailed procedures of accelerate-feedback-control is described in Supplementary Movie 1. It explicitly shows that the output power density varies in response to the change in acceleration^36^. The results demonstrate that the cantilever has excellent acceleration sensitivity. Commonly, as the high-power device, the HEMT operates at the high bias (i.e., the saturation region). The output power (or current) density of the SPD varies significantly in the saturation region, which is much larger than that of the linear region. And thus, the SPD should operate at a large *V*_ds_ (e.g., 15 V) in the saturation region for acceleration-feedback-control. The relationship between different acceleration (1–5 G) and relative output power change (ΔP) is shown in Fig. 6d. As the acceleration increases from 1 to 5 G, the capability to self-regulate output power is enhanced, and ΔP increases from 72.78 W cm^−2^ to 132.89 W cm^−2^ at a large V_ds_ of 15 V while it increases from 5.23 W cm^−2^ to 11.64 W cm^−2^ at a small *V*_ds_ of 5 V. Remarkably, the excellent acceleration sensitivity further demonstrates the feasibility of mechanical stimulation to modulate the output power of the SPD with suitable feedbacks and lay a solid foundation for the development of power devices in the field of AI.

## Discussion

In conclusion, we present a bioinspired SPD for directly controlling output power density in response to the mechanical stimuli, based on the piezotronic effect. Ultra-high values of output power density (W cm^−2^) control under a weak force (mN) control are achieved. The output power density of the SPD increases to 2.72 × 10^3^ W cm^−2^ under an external strain of 16 mN, which also exhibits a good sensitivity. The strain-induced piezoelectric polarization charge can contribute to modifying the E_c_ distribution at the local AlGaN/AlN/GaN heterojunction, and effectively adjust the concentration of 2DEG to tune/control the output current and power density of the SPD. In analogy to the ultimate control capability of the brain in the biological model, the gate voltage bias of the SPD can directly control the output power. Furthermore, the direct, sensitive, and real-time acceleration-feedback-control is achieved in a single chip. This structure combines the advantages of high output power density and programmable gate-control response of AlGaN/AlN/GaN heterojunction HEMT by using the piezoelectric effect of flexible GaN-based cantilevers. The SPD will be very suitable for future AI applications including but not limited to autopilot, intelligent robots, and human-machine interface technologies.

## Methods

### Device fabrication

The SPD was fabricated using III-nitride epitaxial layers by metal-organic chemical vapor deposition (MOCVD) on Si substrate (111) with the 2DEG sheet density of 8 × 10^12^ – 1 × 10^13^ cm^−2^. The epitaxial layer structure consists of AlGaN (30 nm, 30% Al) /AlN (1 nm) / GaN (4.3 μm) / AlGaN buffer layer/Si substrate. The mesa and cantilever patterns were etched using an inductively coupled plasma etching system (ICP, SENTECH SI 500) etch process based on BCl_3_/Cl_2_/Ar and SF_6_/O_2_/Ar. Ti/Al/Ni/Au (20 nm/120 nm/45 nm/55 nm) metal stacks deposition were evaporated by using electron beam evaporation system (Denton Vacuum Explorer 14) and annealed at 850 °C in N_2_ environment for 30 s to form an ohmic contact using a rapid thermal processing system (LABSYS RTP-1200). Ni/Au (80 nm/50 nm) were evaporated for gate metallization to form Schottky contacts. Finally, the ICP-based dry etching was performed by combing the anisotropic/isotropic etching to release the cantilever. The details of the ICP-based dry etching steps are schematically illustrated in Supplementary Fig. 3. Step 1: anisotropic etching of GaN/Si. The photoresist patterned GaN thin film (thickness: 5 μm) was completed etched by using the anisotropic etching recipe (BCl_3_/Cl_2_/Ar: 10 /32/5 sccm; Power: 550 W; Process time: 20 min). Step 2: isotropic etching of Si. The cantilever structure was fabricated with the isotropic etching recipe (SF_6_/O_2_/Ar: 30/5*/*10 sccm; Power: 800 W; Process time: 25 min). The manufactured cantilever had dimensions of 350 × 50 × 5 μm^3^, with the embedded HEMT had a mesa dimension of 27 × 27 μm^2^ and a gate length of 5 μm (see Supplementary Fig. 2 for the detailed process flow).

### Characterizations

The morphology of the SPD was observed by using field-emission scanning electron microscopy (Nova Nano SEM 450) operated at 10 kV. The focused ion beam scanning electron microscope (FIB, FEI Helios NanoLab 600i) is used to focus the ion beam into a tiny size ion beam to bombard the material surface to cut and thin the SPD. The high-resolution transmission electron microscope (HRTEM, TECNAI F20) was then operated at 200 kV and equipped with an energy-dispersive X-ray spectrometer (EDS) detector to characterize the AlGaN/AlN/GaN heterojunctions structure. The corresponding SEAD pattern further suggests the excellent crystallinity of the AlGaN/AlN/GaN heterojunctions. Confocal Raman spectrometer (HORIBA LabRAM HR Evolution) is used to characterize internal strain changes between SPD and HEMT.

### Strain-modulated output characteristics experiments

External strain along *c*-axis was applied on cantilevers by probe station (step moving ~5 μm), as shown in Fig. 3. We bent the cantilever by contacting the cantilever edge using a needle with a tip diameter of 10 μm. By moving the probe downward, a compressive strain was loaded on the cantilevers. The value of strain applied to the cantilever was calculated following the method reported previously by Eliza et al.^37^. The external strain (σ) has been calculated using the formula according to the depression depth of the cantilever as shown in Supplementary Note 1. The bending strain is calculated based on the structural properties of GaN. Keysight B1500A was used to apply voltage to the SPD, and to measure both the current and power of the SPD.

### Acceleration-feedback-control experiments

Keysight B1500A and Keysight LCR E4980A were used for acceleration measurements to collect and record the data. in conjunction with a linear motor. The output power densities at various accelerations including 1, 2, 3, 4, and 5 G were measured.

### Reporting summary

Further information on research design is available in the Nature Research Reporting Summary linked to this article.

## Supplementary information

Supplementary Information

Reporting Summary

Description of Additional Supplementary Files

Supplementary Movie 1

## Data Availability

All data supporting this study and its findings are available within the article and its Supplementary Information or from the corresponding author upon reasonable request.
