# Supplementary material for: Strain-controlled power devices as inspired by human reflex
Source: Nat Commun. 2020 Jan 16;11:326. doi: 10.1038/s41467-019-14234-7 (PMC6965117; doi:10.1038/s41467-019-14234-7)
Supplement: Supplementary file 1 — Supplementary Information [file 41467_2019_14234_MOESM1_ESM.pdf]

# Supplementary Information

## Strain-controlled Power Devices as Inspired by Human Reflex

Shuo Zhang[1,2,#], Bei Ma[3,#], Xingyu Zhou[1,2,#], Qilin Hua[1,2]*, Jian Gong[6], Ting Liu[1,2], Xiao Cui[1,2], Jiyuan Zhu[1,2], Wenbin Guo[1,2], Liang Jing[1,2], Weiguo Hu[1,2,5,]*, and Zhong Lin Wang[1,2,4,5,]*

[1] *CAS Center for Excellence in Nanoscience, Beijing Key Laboratory of Micro-nano Energy and Sensor, Beijing Institute of Nanoenergy and Nanosystems, Chinese Academy of Sciences, Beijing, 100083, China*

[2] *School of Nanoscience and Technology, University of Chinese Academy of Sciences, Beijing 100049, P. R. China*

[3] *Graduate School of Electrical and Electronic Engineering, Chiba University, Chiba 263-8522, Japan*

[4] *School of Materials Science and Engineering, Georgia Institute of Technology, Atlanta, GA 30332-0245, USA.*

[5] *Center on Nanoenergy Research, School of Physical Science and Technology, Guangxi University, Nanning, 530004, China.*

[6] *Estuarine and Coastal Environment Research Center, Chinese Research Academy of Environmental Sciences, Beijing, 100012, P. R. China*

[#]These authors contributed equally to this work

* To whom the correspondence should be addressed.

Email: Qilin Hua (huaqilin@binn.cas.cn), Weiguo Hu (huweiguo@binn.cas.cn),

Zhong Lin Wang (zhong.wang@mse.gatech.edu)

Keywords: bioinspired, power devices, GaN, strain, Piezotronic effect

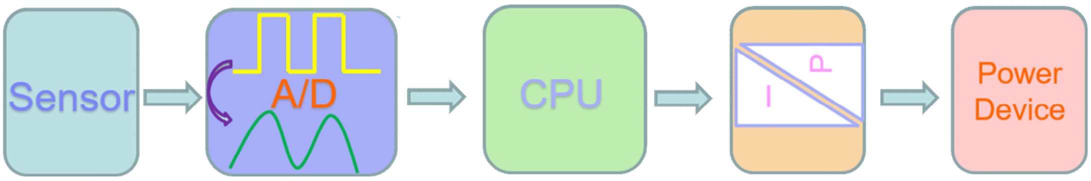


**Supplementary Figure 1 |** Conventional sensor-actuator systems use sensitive components and varistors to convert mechanical signals (displacement, velocity, acceleration, etc.) into electrical signals (voltage, current, etc.) through a series of processes, such as analog-to-digital (A/D) or digital-to-analog (D/A) conversion, strong and weak electrical isolation and CPU control.

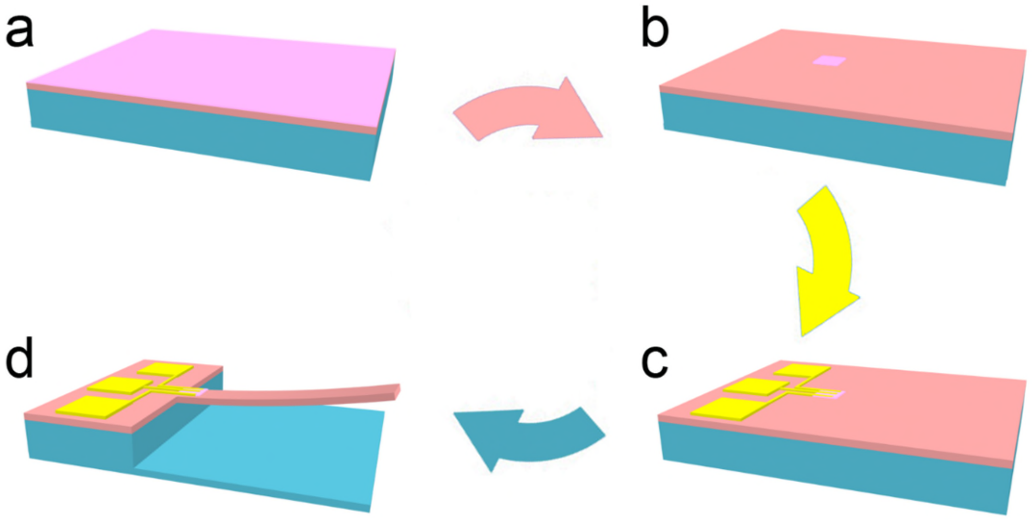


**Supplementary Figure 2** | Fabrication process flow chart of the SPD. **a,** A diced sample with AlGaN/AlN/GaN epilayers grown on Si sample. **b**, The ~300 nm of AlGaN, AlN and GaN mesa etching by $BCl_3/Cl_2/Ar$ based ICP ; **c**, Electron beam deposition of Ti (20 nm)/Al (100 nm)/Ni (45 nm)/Au (55 nm) metal stack and Ni (80 nm)/Au (50 nm) metal stacks to prepare Ohmic contact and Schottky contact electrodes. **d**, Si etching was performed by a gas etching process to release the cantilever and the size of the manufactured cantilever is $350 \times 50 \times 5\ \mu m^3$.

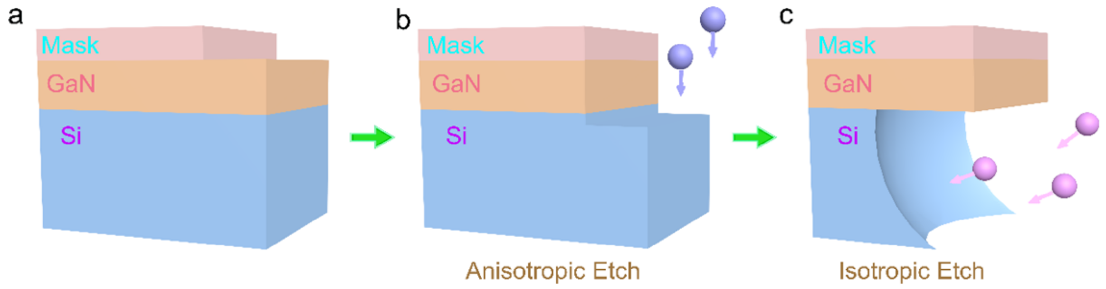


**Supplementary Figure 3** | Etching process flow chart of the SPD. **a**, Patterning the cantilever with positive photo resist. **b**, Fabricating a trench by anisotropic etching of GaN/Si. **c**, Releasing the cantilever structure by isotropic etching of Si.

The details of the ICP-based dry etching steps are described. Step 1: anisotropic etching of GaN/Si. The photoresist patterned GaN thin film (thickness: 5 μm) was completed etched by using the GaN anisotropic etching recipe ($BCl_3/Cl_2/Ar$: 10/32/5 sccm; Power: 550 W; Process time: 20 min). Step 2: Si isotropic etching. The cantilever structure was fabricated with the Si isotropic etching recipe ($SF_6/O_2/Ar$: 30/5/10 sccm; Power: 800 W; Process time: 25 min).

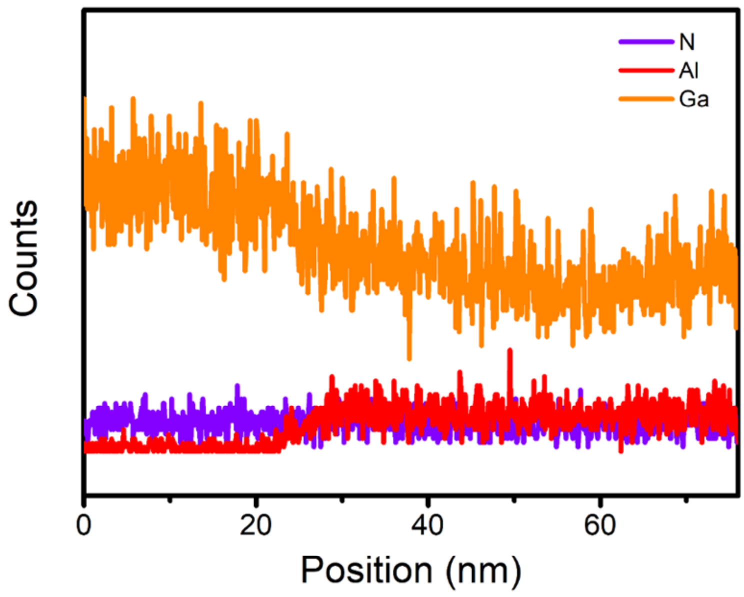


**Supplementary Figure 4 |** EDX line profiles for the element of Ga (orange), Al (red), and N (purple).

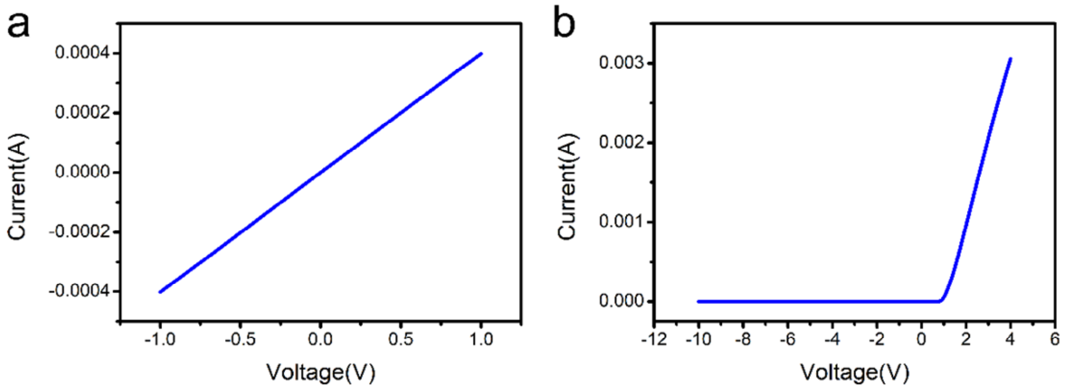


**Supplementary Figure 5 |** I-V characteristics of the SPD. **a**, Ohmic contact curve, and **b**, Schottky contact curve.

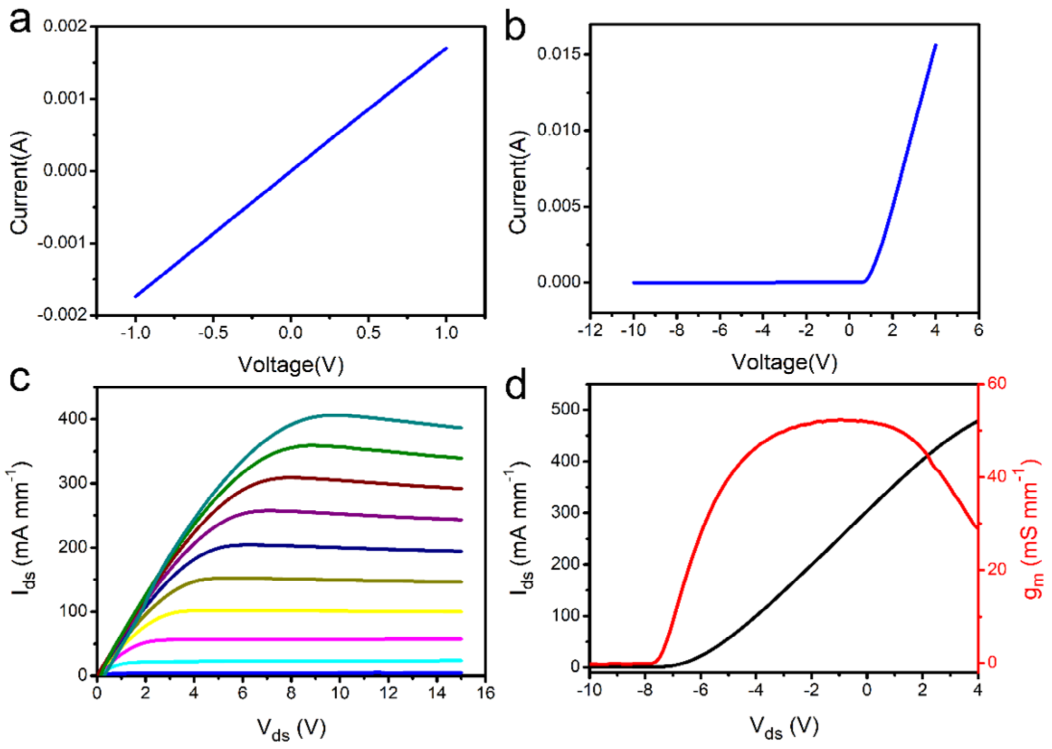


**Supplementary Figure 6** | Measured I-V characteristics of the HEMT (before the dry-etching for the cantilever structure). **a**, Ohmic contact curve. **b**, Schottky contact curves. **c**, $I_{ds}$-$V_{ds}$ curves from bottom to top correspond $V_{gs}$ from -5 V to 1 V, step by 1 V. **d**, $I_{ds}$-$V_{gs}$ characteristic at $V_{ds}$ = 6 V.

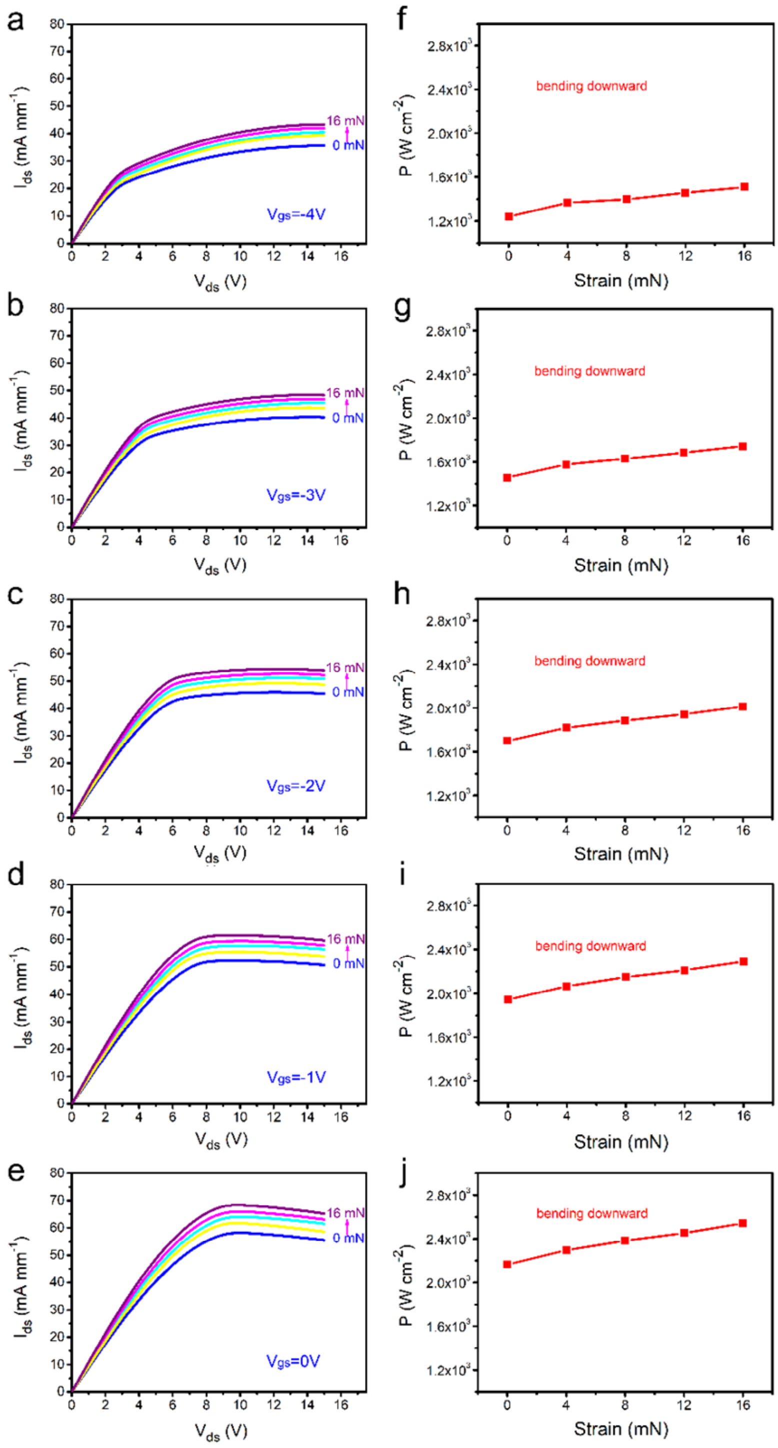


**Supplementary Figure 7** | $I_{ds}$-$V_{ds}$ characteristics of the SPD to various strain at **a**, -4 V, **b**, -3V, **c**, -2V, **d**, -1V and **e**, 0 V biased voltage. Plots of power density sensitivity when gate bias are **f**, -4V, **g**, -3V, **h**, -2V, **i**, -1V, and **j**, 0V under external strain from 0 mN to 16 mN, respectively.

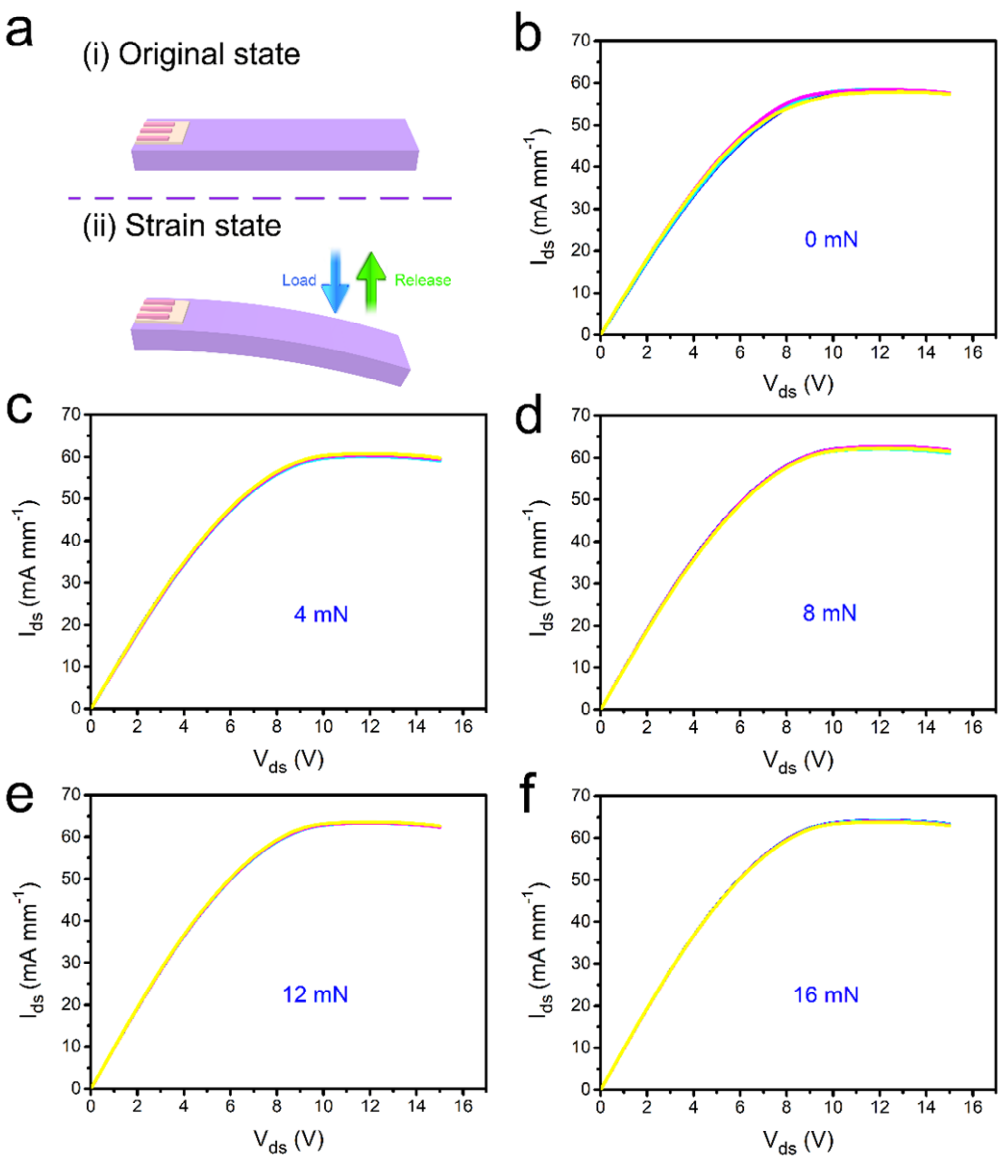


**Supplementary Figure 8** | The reproducibility of the SPD under external strain. **a**, Schematic illustration of the external strain program (load and release). $I_{ds}$-$V_{ds}$ characteristics of the SPD to various strain of **b**, 0 mN, **c**, 4 mN, **d**, 8 mN, **e**, 12 mN and **f**, 16 mN at a $V_{ds}$ bias of 0V, respectively.

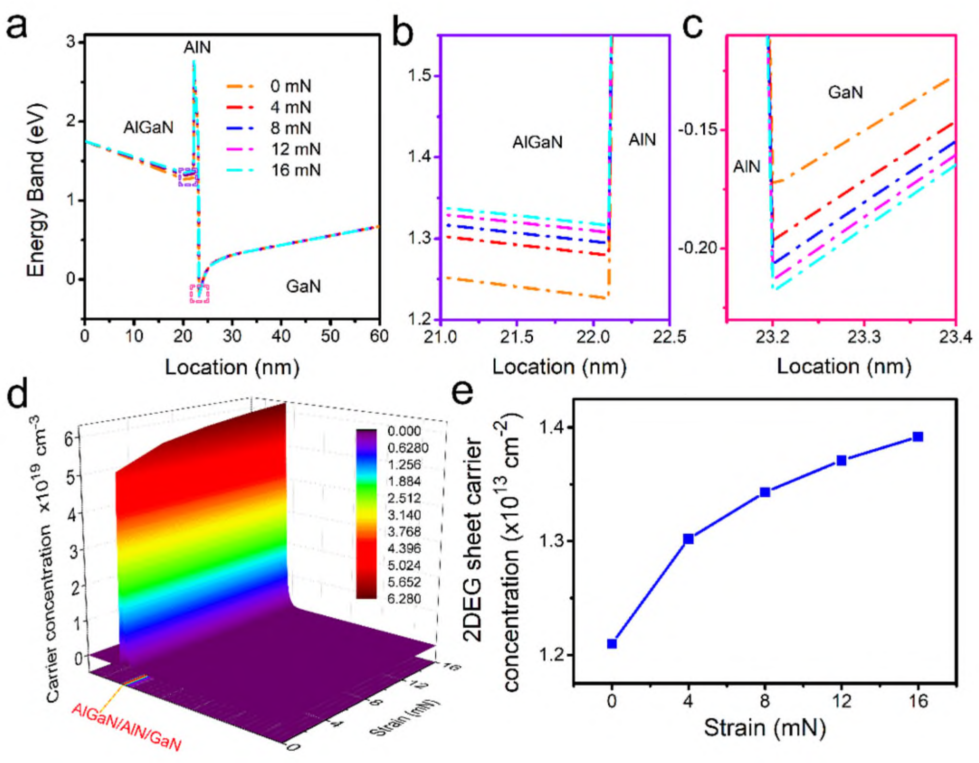


**Supplementary Figure 9 |** The calculation of energy profiles and 2DEG concentration. **a**, The conduction band energy profiles under external strains along *c*-axis of the AlGaN/AlN/GaN heterostructure. The enlarged *E*c at AlGaN/AlN (**b**) and AlN/GaN (**c**) heterojunction as labeled by purple and pink rectangular box in **a**, respectively. **d**, The distribution of carrier concentration in the AlGaN/AlN/GaN heterostructure under external strains. **e**, The 2DEG sheet carrier concentration extracted from **d**.

**Supplementary Note 1**

Equations for calculating external strain (*F*) in the SPD.

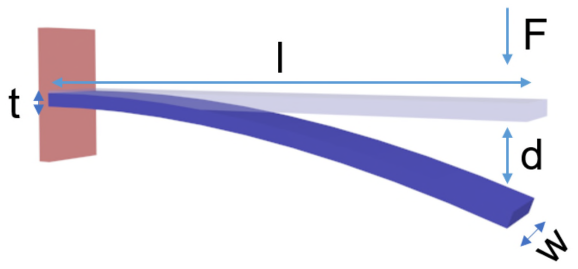


Elastic bending of the SPD system has been carefully studied using Stoney's equation. The depression of the cantilever is used in this work, and the moment of inertia I of the beam can be estimated from the thickness *t* and width *w* of the cantilever beam, as shown in Figure 3c. Both the external strain (*F*) and the resulting vertical displacement (δ) have been calculated from the depression of the cantilever using the following equation, respectively:

$$I = \frac{wt^3}{12} \qquad \text{Equation (1)}$$

$$\delta = \frac{Fl^3}{3EI} \qquad \text{Equation (2)}$$

where *t*, *w*, and *l* are the sample thickness, width and length of the cantilever beam, respectively, *E* is Young's modulus of GaN.

**Supplementary Movie 1.**

The detailed procedures of accelerate-feedback-control are described, and self-regulation of the output power density of the SPD at real-time in response to the acceleration of 1, 2, 3, 4 and 5 G at a $V_{ds}$ of 1 V.
