# Supplementary material for: Strain-controlled power devices as inspired by human reflex
Source: Nat Commun. 2020 Jan 16;11:326. doi: 10.1038/s41467-019-14234-7 (PMC6965117; doi:10.1038/s41467-019-14234-7)
Supplement: Supplementary file 3 — Description of Additional Supplementary Files [file 41467_2019_14234_MOESM3_ESM.pdf]

Description of Additional Supplementary Files

File Name: Supplementary Movie 1
Description: The detailed procedures of accelerate-feedback-control are described, and self-regulation of the output power density of the SPD at real-time in response to the acceleration of 1, 2, 3, 4 and 5 G at a Vds of 1 V.
